# Synthetic co‐culture of autotrophic *Clostridium carboxidivorans* and chain elongating *Clostridium kluyveri* monitored by flow cytometry

**DOI:** 10.1111/1751-7915.13941

**Published:** 2021-10-20

**Authors:** Miriam Bäumler, Martina Schneider, Armin Ehrenreich, Wolfgang Liebl, Dirk Weuster‐Botz

**Affiliations:** ^1^ Institute of Biochemical Engineering TUM School of Engineering and Design Technical University of Munich Boltzmannstr. 15 Garching 85748 Germany; ^2^ Chair of Microbiology TUM School of Life Sciences Technical University of Munich Emil‐Ramann‐Str. 4 Freising Germany

## Abstract

Syngas fermentation with acetogens is known to produce mainly acetate and ethanol efficiently. Co‐cultures with chain elongating bacteria making use of these products are a promising approach to produce longer‐chain alcohols. Synthetic co‐cultures with identical initial cell concentrations of *Clostridium carboxidivorans* and *Clostridium kluyveri* were studied in batch‐operated stirred‐tank bioreactors with continuous CO/CO_2_‐gassing and monitoring of the cell counts of both clostridia by flow cytometry after fluorescence *in situ* hybridization (FISH‐FC). At 800 mbar CO, chain elongation activity was observed at pH 6.0, although growth of *C. kluyveri* was restricted. Organic acids produced by *C. kluyveri* were reduced by *C. carboxidivorans* to the corresponding alcohols butanol and hexanol. This resulted in a threefold increase in final butanol concentration and enabled hexanol production compared with a mono‐culture of *C. carboxidivorans*. At 100 mbar CO, growth of *C. kluyveri* was improved; however, the capacity of *C. carboxidivorans* to form alcohols was reduced. Because of the accumulation of organic acids, a constant decay of *C. carboxidivorans* was observed. The measurement of individual cell concentrations in co‐culture established in this study may serve as an effective tool for knowledge‐based identification of optimum process conditions for enhanced formation of longer‐chain alcohols by clostridial co‐cultures.

## Graphical abstract

Synthetic co‐cultures with identical initial cell concentrations of *Clostridium carboxidivorans* and *Clostridium kluyveri* were studied in batch operated stirred‐tank bioreactors with continuous CO/CO_2_‐gassing and monitoring of the cell counts of both clostridia by flow cytometry after fluorescence *in situ* hybridization (FISH‐FC). At 800 mbar CO, chain elongation activity was observed at pH 6.0, although growth of *C. kluyveri* was restricted. At 100 mbar CO, growth of *C. kluyveri* was improved; however, the capacity of *C. carboxidivorans* to form alcohols was reduced.

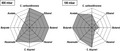

## Introduction

Reducing the concentrations of emissions of greenhouse gases such as CO_2_ is an important criterion for reducing the effect of climate change. In this context, there has been great interest in biological CO_2_‐fixing processes that are able to effectively convert CO_2_ emissions or synthesis gas into multi‐carbon organic chemicals and which may therefore allow for the establishment of a circular carbon economy (Venkata Mohan *et al*., 2016). Synthesis gas (syngas) consists of carbon monoxide (CO), carbon dioxide (CO_2_) and hydrogen gas (H_2_) in addition to inert nitrogen gas (N_2_) and some minor compounds. Syngas is a by‐product of many industrial processes and can also be obtained through the gasification of organic residues (Daniell *et al*., 2012; Molitor *et al*., 2016). Acetogenic bacteria are able to produce chemicals in aqueous media through the autotrophic conversion of CO_2_/H_2_ with a high energetic efficiency of up to 70–90% (Claassens *et al*., 2019). Autotrophic acetogens such as *Clostridium drakei* produce acetate and ethanol as well as 1‐butyrate and 1‐butanol as natural products (Liou *et al*., 2005), whereas autotrophic acetogens such as, on the one hand, *Clostridium autoethanogenum* are also capable of producing 2,3‐butanediol (Köpke *et al*., 2011) and, on the other hand, *Clostridium carboxidivorans*, in some cases, small amounts of 1‐hexanoate/1‐hexanol (Phillips *et al*., 2015). Recombinant acetogens have already been designed to broaden the product spectrum of acetogens (e.g. acetone, isopropanol, 3‐hydroxypropionate, mevalonate, isoprene, farnesene, butanoic acid butyl ester, methyl ethyl ketone and isobutanol) from syngas. However, the yields and product concentrations reported so far have been low (Bengelsdorf *et al*., 2018).

A well‐studied acetogenic bacterium for the conversion of syngas is *C*. *carboxidivorans* (Wan *et al*., 2017), especially because of its ability of forming the medium‐chain fatty acid 1‐hexanoate and the respective alcohol 1‐hexanol (Phillips *et al*., 2015). As common for acetogenic clostridia, batch fermentations can be separated into two phases (Ukpong *et al*., 2012): the acetogenic phase, in which organic acids such as acetate were formed (Datar *et al*., 2004), and the solventogenic phase, in which the organic acids produced are reduced to the corresponding alcohols (Hurst and Lewis, 2010). The acids formed in the acetogenic phase pass through the cell membrane via diffusion due to the decreased external pH compared with the cytoplasm subsequently deprotonate internally and thus disturb the vital proton gradient of the cell over time (Herrero, 1983; Bowles and Ellefson, 1985). A reduction of the fatty acids to the corresponding alcohols in the solventogenic phase leads to an increase of the external pH (Jones and Woods, 1986) and can therefore be seen as a survival mechanism of the cells (Dürre, 2005; Kumar *et al*., 2014).

Higher volumetric productivities can be achieved in continuous gas fermentation processes by connecting two bioreactors with *C. carboxidivorans* in series. In the continuous conversion of CO to produce alcohols (ethanol, 1‐butanol and 1‐hexanol) with pH 6.0 in the first reactor and pH 5.0 in the second reactor, higher space‐time yields than expected were obtained compared with the batch process (increase by a factor of 6 for the alcohols ethanol, 1‐butanol and 1‐hexanol). Surprisingly, the product concentrations in the continuous cascade process were also increased by a factor of three on average (Doll *et al*., 2018). This is due to the unusual property of *C. carboxidivorans* to form about as much ethanol and 1‐butanol as acetate and 1‐butyrate in the acetogenic phase in the first reactor during growth. *Clostridium kluyveri* is able to convert a mixture of acetate and ethanol to 1‐butyrate, 1‐hexanoate (Seedorf *et al*., 2008) and small amounts of 1‐octanoate (Reddy *et al*., 2018). For this purpose, ethanol is first oxidized to form acetyl‐CoA; this is combined with another molecule of acetyl‐CoA to form acetoacetyl‐CoA. By forming different intermediates, analogous to a reverse oxidation, in the first cycle, butyryl‐CoA is formed and coupled with acetate resulting in the release of 1‐butyrate and acetyl‐CoA. If butyryl‐CoA remains in the cycle, hexanoyl‐CoA is formed; this can then be discharged from the cycle as 1‐hexanoate and acetyl‐CoA. During these metabolic chain elongating processes, energy can be conserved by the Rnf complex from reduced ferredoxin because of the exothermic reduction of the intermediates crotonyl‐CoA/2‐hexenoyl‐CoA to butyryl‐CoA/carbonyl‐CoA (Thauer *et al*., 1968; Schoberth and Gottschalk, 1969; Seedorf *et al*., 2008; Steinbusch *et al*., 2011). The elongated fatty acids can, in turn, be reduced to the respective alcohols by acetogenic clostridia, resulting in medium‐chain alcohols with industrial relevance as platform chemicals, fuels or additives to fuels or in the textile, perfume and pharmaceutical industries (Dürre, 2007; Lee *et al*., 2008; Abubackar *et al*., 2011; Fernández‐Naveira *et al*., 2017). *C. kluyveri* has already been used for chain elongation in both defined and undefined co‐cultures (Jiang *et al*., 2018). The first approach of a synthetic co‐culture of *C. autoethanogenum* and *C. kluyveri* was studied in anaerobic shake flasks by Diender *et al*. (2016). Richter *et al*. (2016) described thereupon a co‐culture of *Clostridium ljungdahlii* and *C. kluyveri* wild‐type strains in continuously operated stirred‐tank bioreactor converting syngas (60% CO, 35% H_2_, 5% CO_2_). This produced considerable amounts of 1‐butanol and 1‐hexanol as well as small amounts of 1‐octanol. The pH was found to be an important state variable for this co‐culture to function as it has to favour growth and product formation for both, the acetogen and the chain elongator (Richter *et al*., 2016). Additionally, the proportion of ethanol to acetate formed by *C. ljungdahlii* is pH dependent (Abubackar *et al*., 2016). While *C. carboxidivorans* is able to produce 1‐butanol and 1‐hexanol directly from syngas, the production rates, product concentrations and selectivities achieved are low compared with acetate/ethanol (Ukpong *et al*., 2012; Richter *et al*., 2016; Doll *et al*., 2018). As *C. carboxidivorans* is able to produce butyrate/1‐butanol and hexanoate/1‐hexanol directly from CO/CO_2_ in contrast to *C. autoethanogenum* and *C. ljungdahlii*, the combination of an acetogen, which is able to produce (small amounts of) C4 and C6 acids/alcohols with a chain elongating bacterium, seems to be very prospective compared with the already reported combination. Thus, a synthetic co‐culture of *C. carboxidivorans* producing acetate/ethanol as well as 1‐butyrate/1‐butanol and 1‐hexanoate/1‐hexanol from CO and *C. kluyveri* converting the main products acetate/ethanol to 1‐butyrate and 1‐hexanoate, which is directly reduced to 1‐butanol and 1‐hexanol by *C. carboxidivorans*, is a promising approach to broaden the product spectrum of syngas fermentation so far not studied.

Previous studies on co‐cultivations of acetogens with chain elongating bacteria such as *C. kluyveri* published so far are undefined with respect to the individual changes in cell counts of the two species in co‐culture. This paper deals with the establishment of a synthetic autotrophic co‐culture of *C*. *carboxidivorans* and *C*. *kluyveri* in batch‐operated stirred‐tank bioreactors with continuous CO/CO_2_‐gassing and monitoring of the cell counts of both microorganisms with flow cytometry after individual fluorescence *in situ* hybridization of the cells. Research question is to identify changes in batch process performances of the co‐culture with respect to product formation patterns and growth of the two strains in co‐culture monitored by flow cytometry using the designed probes in comparison with pure cultures of *C. carboxidivorans* applying two examples, one with low and one with high initial partial pressure of CO.

## Results and discussion

### Individual batch processes with *C. kluyveri* and *C. carboxidivorans*

Initially, individual reference batch processes were carried out in fully controlled stirred‐tank bioreactors with continuous gas supply. *C. kluyveri* cells were grown heterotrophically at pH 6.8 (Barker and Taha, 1941), and a gas mixture of 80% N_2_ and 20% CO_2_ (Tomlinson and Barker, 1954) with initial acetate and ethanol concentrations of 6.0 g l^−1^ and 15.0 g l^−1^, respectively. *C. carboxidivorans* cells were grown autotrophically with a gas mixture of 80% CO and 20% CO_2_ at pH 6.0. The same media and initial cell dry weight concentrations of *c*_X,0_ = 0.05 g l^−1^ were applied.

**Fig. 1:**
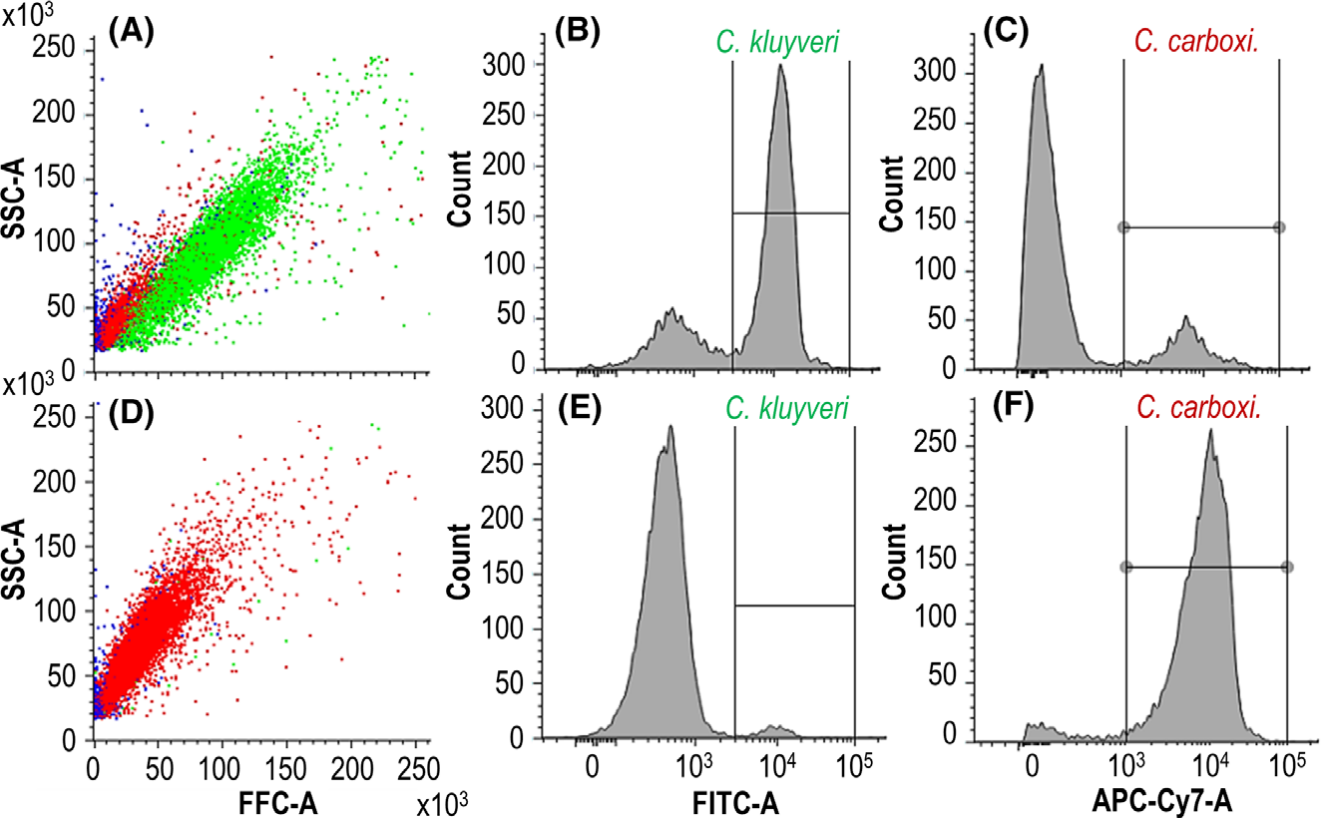
Two samples with 75% *C. kluyveri* and 25% *C. carboxidivorans* (A–C) and 1.5% *C. kluyveri* and 98.5% *C. carboxidivorans* (D–F) cells were mixed with the strain‐specific oligonucleotide probes, and the resulting cell numbers were measured with flow cytometry. (A + D) Scattergrams with the intensity of both fluorescence signals (*C. kluyveri* in green and *C. carboxidivorans* in red). Blue dots indicated other, non‐labelled particles. The axes show the intensity of the fluorescence signal of the side scatter (SSC) vs. the forward scatter (FSC) (arbitrary units). (B + E) Histograms with the cell counts of *C. kluyveri* vs. intensity of the FITC‐fluorescence signal excited by a blue laser at 488 nm and detected using the 527/32 nm band‐pass filter (FITC) manually gated between a fluorescence intensity of 4 × 10^4^–2 × 10^5^. (C + F) Histograms with the cell counts of *C. carboxidivorans* vs. intensity of the Cy5‐fluorescence signal excited by red laser at 640 nm and detected by using the 660/10 nm band‐pass filter (APC‐Cy7) manually gated between a fluorescence intensity of 10^3^ and 10^5^ (A = Area).

Samples for FISH‐labelling and measurement of the cell counts with flow cytometry were withdrawn after 24 h at final CDW concentrations of 0.4 g l^−1^ *C. carboxidivorans* and 0.8 g l^−1^ *C. kluyveri* and stored individually in 50% EtOH/PBS solution at −20°C. Varying ratios of *C. carboxidivorans* and *C. kluyveri* cells were mixed before labelling with the strain‐specific oligonucleotide probes. Fig. 1 shows the results of a mixture of 25% *C. carboxidivorans* and 75% *C. kluyveri* as well as 98.5% *C. carboxidivorans* and 1.5% *C. kluyveri* measured with flow cytometry as examples. Blue dots indicate other, non‐labelled events like remaining particles of the PBS buffer in the sample as well as cell debris that cannot be mislabelled as viable cells as an intact rRNA is the aim for the probe. The relative ratio of the cell counts was 24.3 ± 2.3% *C. carboxidivorans* and 76.5 ± 1.9% *C. kluyveri* (Fig. 1B and C) and 95.6 ± 3.1% *C. carboxidivorans* and 1.7 ± 1.2% *C. kluyveri* (Fig. 1E and F). Deviations are caused by additional particles from PBS wrongly assigned to a cell population (total > 100%) and if cells are not assigned to any population (total < 100%). The populations of *C. carboxidivorans* shown in red seem to vary in cell size depending on the amount of cells applied to the analysis (25% vs. 98.5% percentage of cells). This is most likely due to the fact that the microorganisms may tend to aggregate with increasing cell number which acts like an apparent increase in cell size. Cell aggregation does not pose a problem for determining cell ratio as in our studies, but should be carefully monitored if single‐cell recovery by fluorescence‐activated cell sorting (FACS) is the goal (Haroon *et al*., 2013). Scattergrams (FSC vs. SSC) of both strains grown as pure cultures show nearly the same cell size (Fig. S3) thus proving the necessity for FISH‐FC for determination of *C. kluyveri* and *C. carboxidivorans* cells as a discrimination between both strains is not possible based solely on cell distributions. Within the estimation error of biological triplicates, this demonstrates the successful quantification of the ratio of the two microorganisms by *in solution* FISH labelling and flow cytometry.

**Fig. 2:**
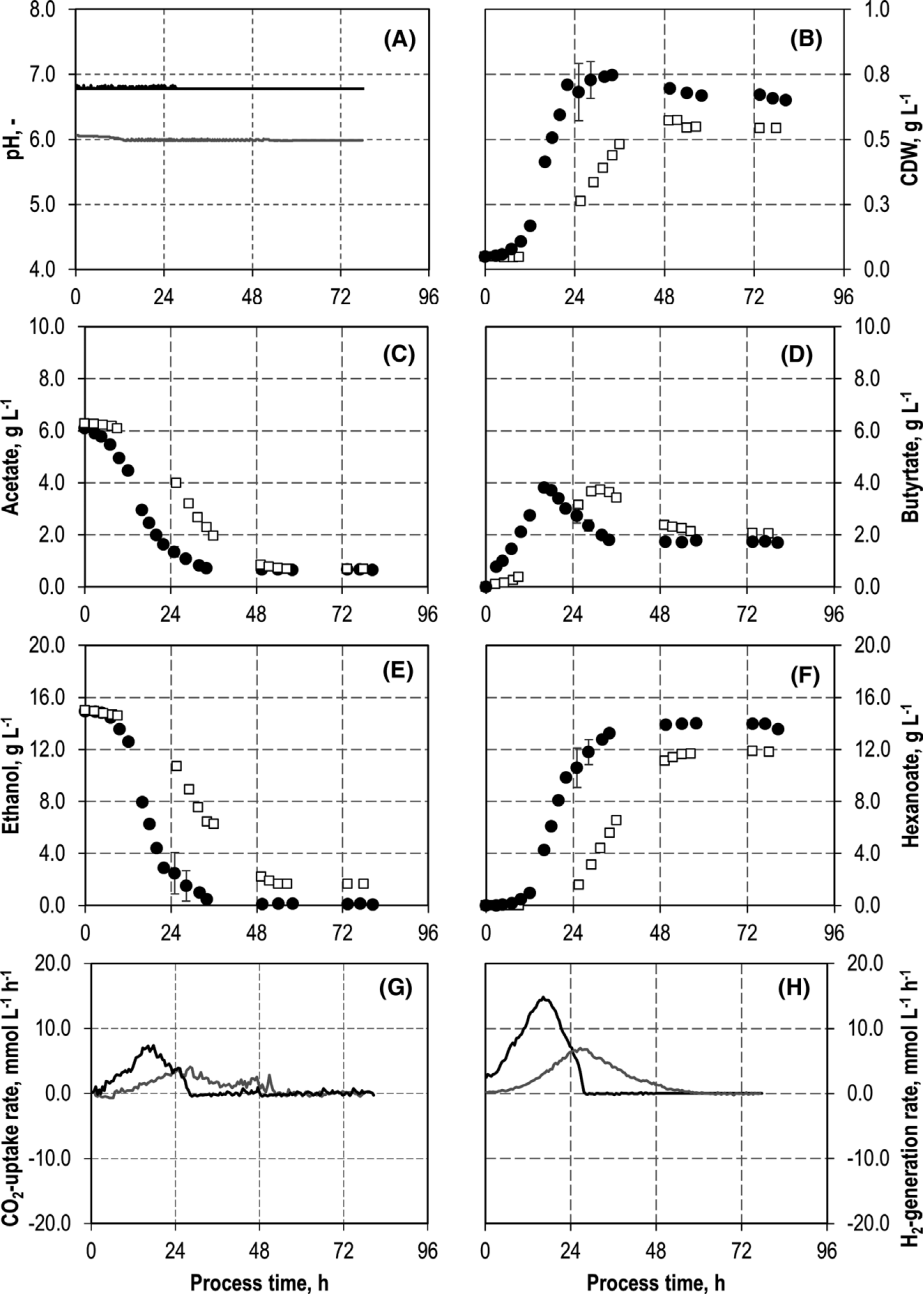
Batch cultivations of *C. kluyveri* in fully controlled stirred‐tank bioreactors at pH 6.8 (black dots and black line) and pH 6.0 (white squares and grey line). A. pH. B. Cell dry weight concentrations (CDW) of *C. kluyveri*. C. Acetate concentrations. D. Butyrate concentrations. E. Ethanol concentrations. F. Hexanoate concentrations. G. Carbon dioxide uptake rate. H. Hydrogen uptake rate (*T* = 37°C; *PV* ^−1^ = 11.7 W l^−1^; *F*_gas_ = 5 l h^−1^ (80:20 N_2_:CO_2_)).

**Fig. 3:**
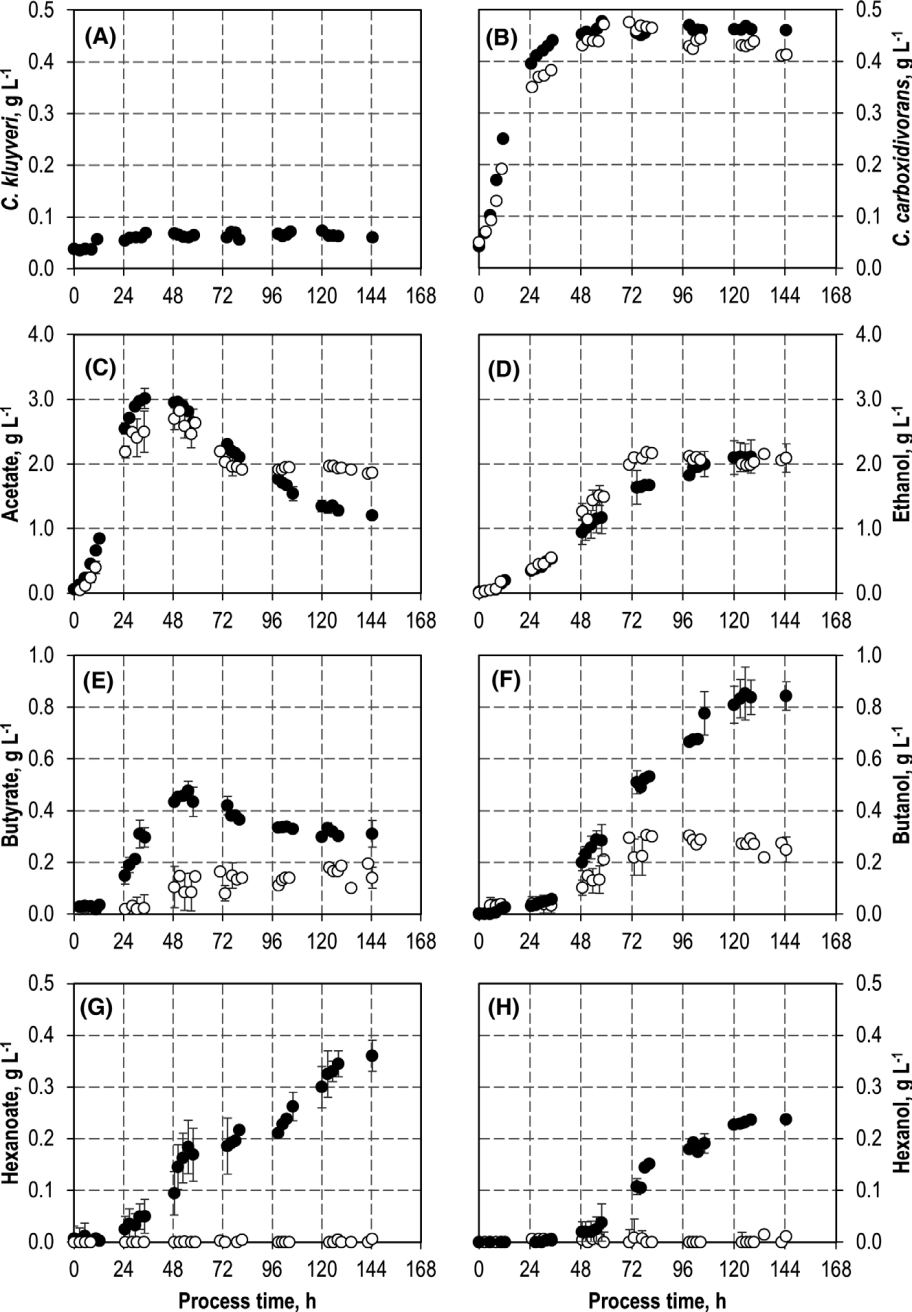
Autotrophic syngas conversion with a co‐culture of *C. carboxidivorans* and *C. kluyveri* (black) compared to a mono‐culture of *C. carboxidivorans* (white) in batch‐operated stirred‐tank bioreactors with continuous gassing with an initial CO partial pressure *p*_CO,in_ = 800 mbar. A. CDW concentration of *C. kluyveri*. B. CDW concentrations of *C. carboxidivorans*. C. Acetate concentrations. D. Ethanol concentrations. E. Butyrate concentrations. F. Butanol concentrations. G. Hexanoate concentrations. H. Hexanol concentrations. Error bars indicate the deviation of the minimum and maximum value from the mean value of two independent batch processes. (*T* = 37°C; pH = 6.0; *P* *V* ^−1^ = 11.7 W l^−1^; *F*_gas_ = 5 l h^−1^ (80:20 CO:CO_2_)).

Batch growth of both strains should be possible and in the same order of magnitude in mono‐culture applying the same reaction conditions in terms of medium composition, temperature and pH to make sure that balanced growth and the formation of intermediates and products will have a high probability to take place in co‐culture. The two individual batch processes with *C. kluyveri* (Fig. 2) and *C. carboxidivorans* (Fig. 3) showed a higher maximum CDW concentration with *C. kluyveri* (0.75 g l^−1^ compared with 0.45 g l^−1^ with *C. carboxidivorans*) after 48 h, thus indicating a significantly lower growth rate and biomass yield with autotrophically grown *C. carboxidivorans*. 14.0 g l^−1^ hexanoate and 2.0 g l^−1^ butyrate were produced with *C. kluyveri* after 48 h, whereas *C. carboxidivorans* produced 2.9 g l^−1^ acetate, 2.2 g l^−1^ ethanol, 0.2 g l^−1^ butyrate and 0.3 g l^−1^ butanol within a process time of 144 h. The concentrations of hexanoate and hexanol were below the detection limit.

This clearly demonstrates the lower metabolic activity of autotrophically grown *C. carboxidivorans* at an optimum pH of 6.0 compared with heterotrophically grown *C. kluyveri* at an optimum of pH 6.8 in batch processes with the same initial biomass concentrations. In order to reduce the growth rate of *C. kluyveri* and to enable co‐cultivation with *C. carboxidivorans,* the pH was reduced to pH 6.0 and the results of the batch process are compared with the batch process at pH 6.8 (Fig. 2).

CDW concentrations of *C. kluyveri* were reduced by 20% to 0.6 g l^−1^ after 48 h at pH 6.0 compared with pH 6.8 (0.75 g l^−1^). A reduction by 40% would have been necessary to achieve the same CDW concentration as for autotrophically grown *C. carboxidivorans*. Acetate and ethanol consumption were delayed at pH 6.0. However, finally the acetate consumption was the same as with pH 6.8, whereas ethanol was not fully consumed. Chain elongation by *C. kluyveri*, including the reconsumption of butyrate, was observed in both batch processes. The final butyrate concentration was independent of the pH, whereas the final hexanoate concentration was higher at pH 6.8 (14.0 g l^−1^ compared with 11.9 g l^−1^).

As carbon dioxide is considered as essential carbon source for cell proliferation of *C. kluyveri,* and hydrogen occurs as an additional product of the chain elongation process, uptake rates of CO_2_ and generating rates of H_2_ may serve as indicators for metabolic activity of *C. kluyveri*. Metabolic activities of *C. kluyveri* were reduced at pH 6.0. The maxima in the CO_2_ uptake and the H_2_ generation rates were reduced by around twofold to 6.8 mmol l^−1^ h^−1^ H_2_ (compared with 14.8 mmol l^−1^ h^−1^) and 3.3 mmol l^−1^ h^−1^ CO_2_ (compared with 6.5 mmol l^−1^ h^−1^), respectively (Fig. 2G and H). On the other hand, the reduced pH resulted in nearly the doubling of the process time with CO_2_ uptake and the H_2_ generation. Carbon balances were closed with carbon recoveries of 98% and 99% at pH 6.0, and pH 6.8, respectively.

The substrate consumption rates of *C. kluyveri* at pH 6.0 were 0.2 g l^−1^ h^−1^ acetate and 0.4 g l^−1^ h^−1^ ethanol within 48 h. Because the product formation rates of autotrophically grown *C. carboxidivorans* were lower (0.15 g l^−1^ h^−1^ acetate and 0.05 g l^−1^ h^−1^ ethanol within 48 h, see Fig. 3), no accumulation of ethanol and acetate should be expected in co‐cultivation of both strains at pH 6.0. Although it is likely that the product concentrations and product formation rates in co‐cultivation of both organisms will differ from the results obtained in pure cultures, thus necessitating an adjustment of the thermodynamic balance as shown in previous experiments (Diender *et al*., 2019), batch processes at pH 6.0 should provide suitable conditions for co‐cultivation of both strains with an initial ratio of 1:1.

### Autotrophic co‐cultivation of *C. carboxidivorans* and *C. kluyveri*

**Fig. 4:**
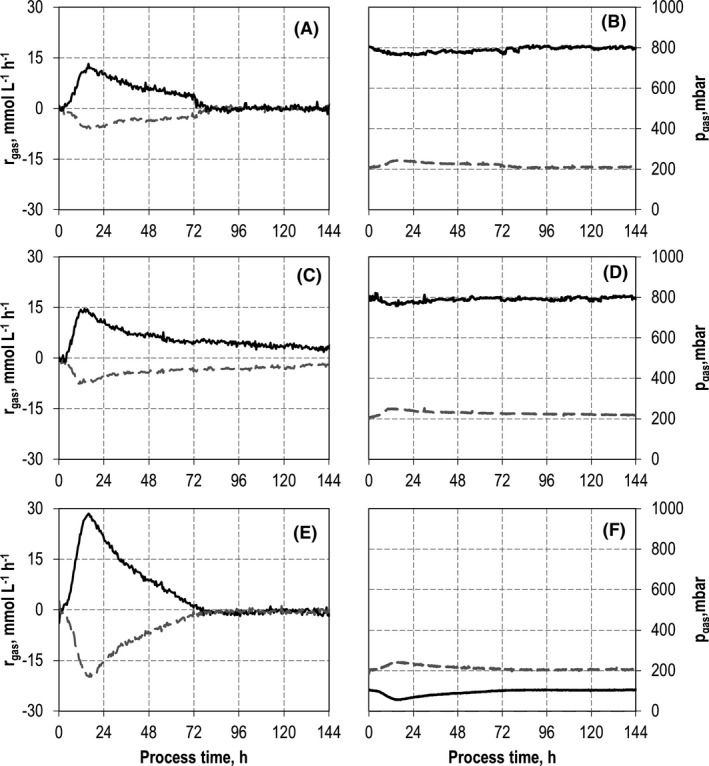
Gas consumption rates and gas partial pressures of CO (black solid line), CO_2_ (dark grey dotted line) of autotrophic batch processes in continuously gassed stirred‐tank bioreactors with *C. carboxidivorans* with an initial CO partial pressures *p*_CO,in_ = 800 mbar (A, B), with a co‐culture of *C. carboxidivorans* and *C. kluyveri* with an initial CO partial pressures *p*_CO,in_ = 800 mbar (C, D) and with a co‐culture with initial CO partial pressures *p*_CO,in_ = 100 mbar (E, F); (*T* = 37°C; pH = 6.0; *P* *V* ^−1^ = 11.7 W l^−1^; *F*_gas_ = 5 l h^−1^).

An initial co‐cultivation process was performed with initial cell concentrations of 0.5 g l^−1^ *C. carboxidivorans* and 0.5 g l^−1^ *C. kluyveri* at pH 6.0 with continuous gas supply (80% CO, 20% CO_2_). The results are compared to a batch process with *C. carboxidivorans* (Figs 3 and 4). Both batch processes were reproduced and showed identical process performances within the estimation error based on biological duplicates.

Growth of *C. carboxidivorans* started immediately after inoculation of the co‐culture. A CDW concentration of 0.44 ± 0.02 g l^−1^ was measured after 48 h showing no significant difference to the batch process with only *C. carboxidivorans*. In contrast to *C. carboxidivorans*, growth of *C. kluyveri* was considerably reduced in the co‐culture. A CDW concentration of 0.07 ± 0.01 g l^−1^ *C. kluyveri* was observed after 48 h.

Acetate formation was the same after 48 h compared with a batch process with a mono‐culture of *C. carboxidivorans,* whereas the ethanol concentration was reduced in the co‐culture after 48 h (0.9 ± 0.1 g l^−1^ in co‐culture compared with 1.2 ± 0.1 g l^−1^). Finally, nearly the same ethanol concentration of 2.1 ± 0.2 g l^−1^ was measured in the co‐culture after 144 h. However, the final acetate concentration was clearly reduced in co‐culture compared with the batch process with *C. carboxidivorans* (1.2 ± 0.02 g l^−1^ in co‐culture compared with 1.9 ± 0.01 g l^−1^) showing acetate and ethanol consumption by *C. kluyveri* despite the low CDW concentrations of *C. kluyveri* in co‐culture.

As a consequence, butyrate and hexanoate concentrations were strongly enhanced in co‐culture after 48 h with 0.43 g l^−1^ butyrate in co‐culture compared with 0.12 ± 0.03 g l^−1^, and 0.10 ± 0.02 g l^−1^ hexanoate compared with 0 g l^−1^, respectively. Reconsumption of butyrate and hexanoate by both strains resulted in decreasing concentrations of butyrate; however, hexanoate formation by *C. kluyveri* exceeded reconsumption as can be seen by the steadily increasing hexanoate concentration until the end of the batch process. The final butanol concentration of 0.8 ± 0.05 g l^−1^ in co‐culture was increased by a factor of 3.1 compared with the batch process with a mono‐culture of *C. carboxidivorans*. Hexanol was produced only in co‐culture, resulting in 0.24 g l^−1^ after 144 h.

For *C. carboxidivorans*, CO consumption was prolonged in the co‐culture compared with the mono‐culture (Fig. 4A and C). In total, 820 mmol CO were consumed by *C. carboxidivorans* in co‐culture compared with 477 mmol CO with the mono‐culture (increase by a factor of 1.7). The partial pressures of both gases were not considerably reduced in the stirred‐tank reactors because of the low biomass concentrations and gassing in excess.

Carbon balances were closed within the estimation error based on biological duplicates with carbon recoveries of 97% with the co‐culture and 97% with the mono‐culture of *C. carboxidivorans*, respectively.

Despite the low CDW concentrations of *C. kluyveri* in the co‐culture with a ratio of 13% *C. kluyveri* to 87% *C. carboxidivorans* after 48 h, chain elongation by *C. kluyveri* and reduction of butyrate and hexanoate by *C. carboxidivorans* altered the process performance of the co‐culture considerably compared with a batch process with *C. carboxidivorans* under the same reaction conditions. Final C4 and C6 product concentrations were increased at the expense of the C2 products, mainly acetate (Fig. 3). One reason for this remarkable chain elongation activity of *C. kluyveri* at low CDW concentrations in co‐culture may be that chain elongation becomes more efficient at the expense of biomass generation in order to compensate decreased ATP production (González‐Cabaleiro *et al*., 2013; Gildemyn *et al*., 2017). Hexanoate production by *C. carboxidivorans* would be possible at reduced pH (Doll *et al*., 2018) but was not observed at pH 6.0 in mono‐culture (Fig. 3).

Compared with the results of the batch studies with mono‐cultures of *C*. *kluyveri* and *C. carboxidivorans* at pH 6.0, growth of *C. kluyveri* was strongly reduced in co‐culture. Two effects may have caused this. First, ethanol and acetate need to be produced autotrophically by *C. carboxidivorans*, and the ratio and the initial concentrations of the C2 products are low at the start of the process; this may result in low initial growth of *C. kluyveri*. Second, growth of *C. kluyveri* may be inhibited by high CO concentrations in the liquid phase. CO inhibition of *C. kluyveri* at increased CO partial pressures has already been observed (Diender *et al*., 2016). CO inhibition seems to be likely at the beginning of the batch process at low CDW concentrations of *C. carboxidivorans*.

**Fig. 5:**
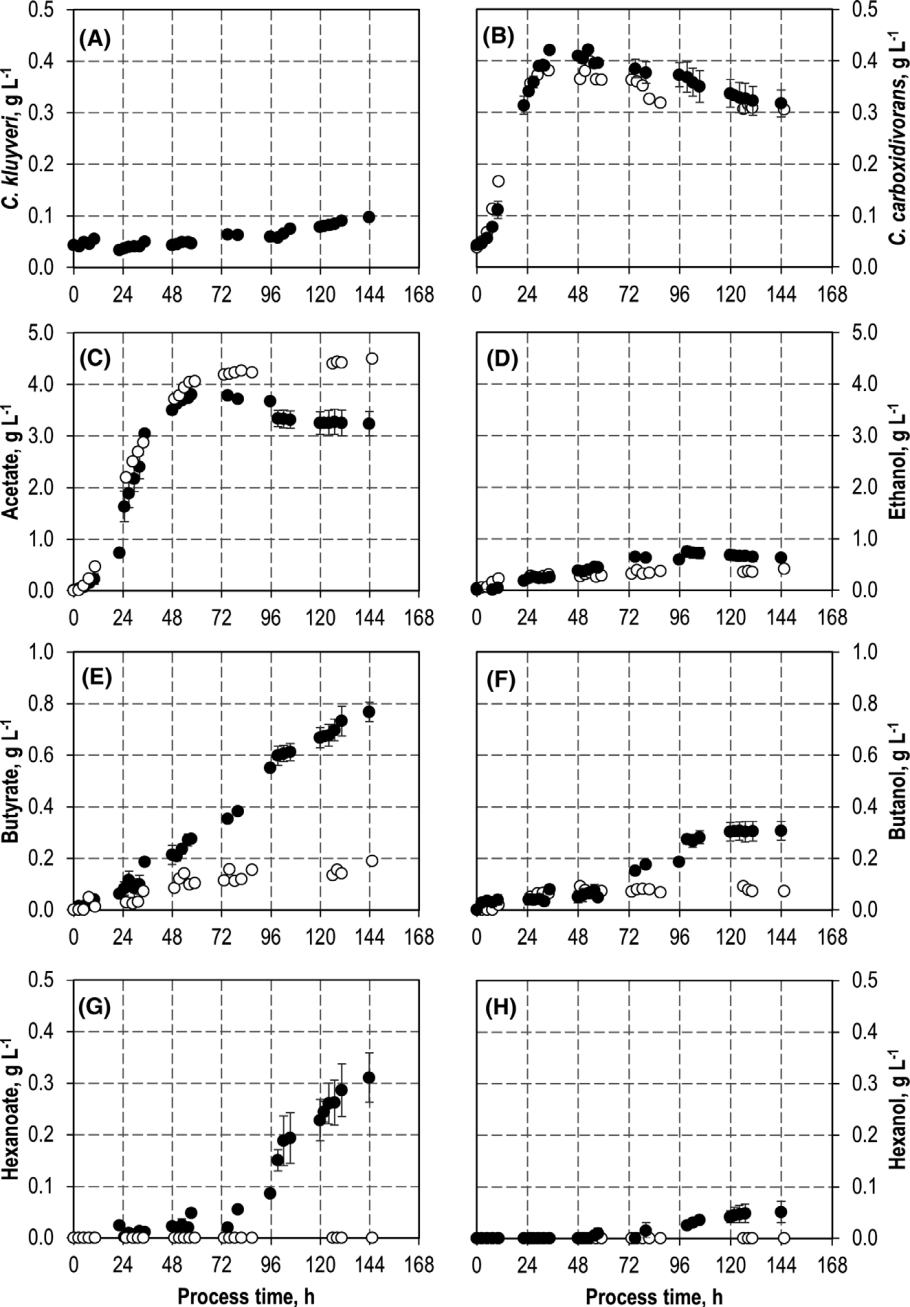
Autotrophic syngas conversion with a co‐culture of *C. carboxidivorans* and *C. kluyveri* (black) and a mono‐culture of *C. carboxidivorans* (white) in batch‐operated stirred‐tank bioreactors with continuous gassing with an initial CO partial pressures *p*_CO,in_ = 100 mbar. A. CDW concentration of *C. kluyveri*. B. CDW concentrations of *C. carboxidivorans*. C. Acetate concentrations. D. Ethanol concentrations. E. Butyrate concentrations. F. Butanol concentrations. G. Hexanoate concentrations. H. Hexanol concentrations. Error bars indicate the deviation of the minimum and maximum value from the mean value of two independent batch processes. (*T* = 37°C; pH = 6.0; *P* *V* ^−1^ = 11.7 W l^−1^; *F*_gas_ = 5 l h^−1^ (10:20 CO:CO_2_)).

Thus, a second co‐cultivation was performed with a reduced inlet CO partial pressure (10% CO, 20% CO_2_ and 70% N_2_) without any other changes. The results are compared to a batch process with *C. carboxidivorans* applying the same inlet gas composition (Fig. 5). The resulting partial pressures in the exhaust gas as well as the consumption rates of CO and CO_2_ are shown in Fig. 4E and F. Both batch processes were reproduced. No changes in process performances were observed within the estimation error based on biological duplicates.

Growth of *C. carboxidivorans* started immediately after inoculation. After 48 h, a CDW concentration of 0.41 g l^−1^ was reached. Afterwards, a constant decay of *C. carboxidivorans* was observed in co‐culture. This was comparable with the decay of the mono‐culture. Finally, the CDW concentration of *C. carboxidivorans* was reduced by more than 30% to 0.3 g l^−1^.

In contrast to *C. carboxidivorans*, no significant growth of *C. kluyveri* was observed within the first 48 h of the co‐culture. Growth of *C. kluyveri* started after this prolonged lag‐phase and showed a constant increase in CDW concentrations reaching a CDW concentration of 0.12 g l^−1^ after 144 h.

Decreasing CDW concentrations of *C. carboxidivorans* at low CO partial pressures in mono‐culture and in co‐culture can be explained by the increased acetate and butyrate concentrations. Acid concentrations of 4 g l^−1^ acetate and 2 g l^−1^ butyrate were reported to show a 50% reduction in maximum CDW concentrations of *C. carboxidivorans* in batch processes with an initial pH 6.0 without further pH control (Zhang *et al*., 2016) because undissociated acids diffuse into the cell and dissociate internally with negative effects on the ion gradient necessary for ATP regeneration (Dürre, 2005). At pH 6.0 throughout the batch process, this effect may not be pronounced as reported because the acid concentrations are reduced compared with the acid anions.

Acetate and ethanol formation were the same after 48 h compared to the batch process with a mono‐culture of *C. carboxidivorans,* whereas ethanol concentration was slightly reduced in the co‐culture after 48 h (0.3 g l^−1^ in co‐culture compared with 0.4 g l^−1^ in mono‐culture). Despite the non‐growth of *C.kluyveri* within the first 48 h, butyrate formation was observed from the beginning and hexanoate formation started after 72 h, showing chain elongation activity by *C. kluyveri*. As a result, butyrate and hexanoate concentrations were increased in co‐culture resulting in 0.77 ± 0.04 g l^−1^ butyrate compared with 0.19 g l^−1^ in the mono‐culture of *C. carboxidivorans* after 144 h, and 0.31 ± 0.05 g l^−1^ hexanoate compared with 0 g l^−1^, respectively. With the exception of butanol, no significant increased formation of alcohols by *C. carboxidivorans* was observed in the co‐culture with 100 mbar CO in the inlet gas phase.

Surprisingly, the maximum CO consumption rate of *C. carboxidivorans* was doubled compared with the process with 80% CO and 20% CO_2_ (Fig. 4A and E). Finally, 1013 mmol CO was consumed by the co‐culture at reduced CO and CO_2_ concentrations in the inlet gas phase compared with 820 mmol CO with 80% CO. Because of the low inlet partial pressure of CO (100 mbar), the partial pressure of CO in the stirred‐tank reactor was considerably reduced to 58 mbar at the minimum. Carbon balances were solely closed within the estimation error based on biological duplicates with carbon recoveries of 96% with the co‐culture. For unknown reasons, only 93% of the carbon was recovered with the mono‐culture of *C. carboxidivorans*.

**Fig. 6:**
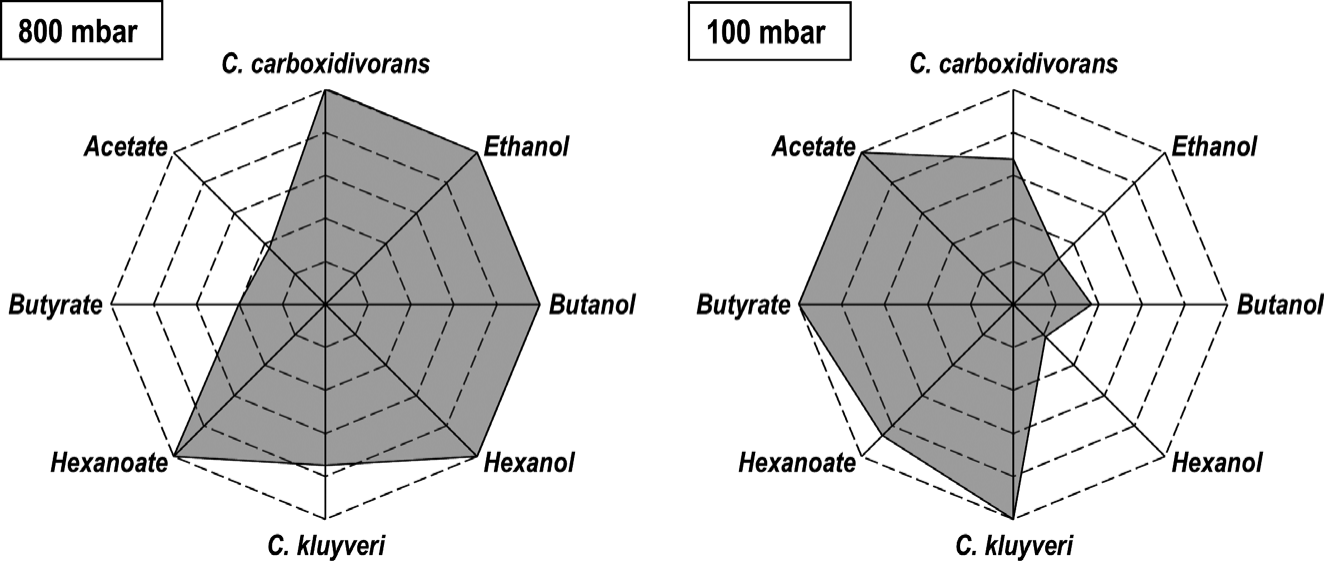
Graphical representation of the relative final CDW concentrations of *C. carboxidivorans* and *C. kluyveri* and the relative final concentrations of alcohols and organic acids during autotrophic syngas conversion with a co‐culture of *C. carboxidivorans* and *C. kluyveri* in batch‐operated stirred‐tank bioreactors with continuous gassing with initial CO partial pressures of *p*_CO,in_ = 800 mbar and *p*_CO,in_ = 100 mbar. (*T* = 37°C; pH = 6.0; *P* *V* ^−1^ = 11.7 W l^−1^; *F*_gas_ = 5 l h^−1^; *t* = 144 h).

The relative final product and biomass concentrations of the batch processes with co‐cultures of *C. carboxidivorans* and *C. kluyveri* are shown graphically in Fig. 6 comparing the process performances at high CO concentrations (800 mbar CO in the inlet gas phase) with low CO concentrations (100 mbar CO). The highest final alcohol and hexanoate concentrations were measured at high CO partial pressure, whereas the concentrations of the organic acids acetate and butyrate were highest at low CO partial pressure. The final butyrate concentration was increased by a factor of 2.5 at low CO partial pressure (Table 1). The acetate to ethanol ratio or availability in the co‐culture has a significant effect on the product spectrum of *C. kluyveri* because higher acetate to ethanol ratios result in increased butyrate production and reduced hexanoate production (Bornstein and Barker, 1948; Weimer and Stevenson, 2012; Yin *et al*., 2017). Butyryl‐CoA is increasingly converted to butyrate by butyryl‐CoA:acetate CoA transferase, thereby resulting in a decreased availability of butyryl‐CoA for the formation of hexanoyl‐CoA (Bielzer, 2019). A consequence of the shifted acetate to ethanol ratio as a result of the decreased CO inlet partial pressure was therefore a decreased chain length of the organic acids produced (see Table 1).

**Table 1:** Maximum specific growth rates of *C. carboxidivorans* as well as the final product concentrations and alcohol‐to‐acid ratios after 144 h measured in autotrophic mono‐cultures of *C. carboxidivorans* and co‐cultures of *C. carboxidivorans* and *C. kluyveri* in pH‐controlled batch processes with continuous gassing in stirred‐tank reactors with 100 or 800 mbar CO in the inlet gas phase

*p*_CO,in_	800 mbar		100 mbar	
	Mono‐culture	Co‐culture	Mono‐culture	Co‐culture
*µ*_max_, h^−1^	0.16	0.15	0.17	0.15
CDW*_C. carboxidivorans_*, g l^−1^	0.41	0.47	0.31	0.32
CDW*_C. kluyveri_*, g l^−1^	–	0.06	–	0.12
Acetate, g l^−1^	1.87 ± 0.01	1.20 ± 0.02	4.50	3.24 ± 0.2
Butyrate, g l^−1^	0.14 ± 0.04	0.31 ± 0.05	0.19	0.77 ± 0.04
Hexanoate, g l^−1^	0.00	0.36 ± 0.03	0.00	0.31 ± 0.07
Ethanol, g l^−1^	2.09 ± 0.03	2.09 ± 0.20	0.41	0.63 ± 0.08
Butanol, g l^−1^	0.25 ± 0.05	0.84 ± 0.06	0.07	0.32 ± 0.06
Hexanol, g l^−1^	0.00	0.24 ± 0.01	0.00	0.05 ± 0.02
EtOH/Ac, g g^−1^	1.12	1.74	0.09	0.19
ButOH/But, g g^−1^	1.77	2.73	0.39	0.40
HexOH/Hex, g g^−1^	–	0.66	–	0.16

Errors indicate the deviation of the minimum and maximum value from the mean value of two independent batch processes (*T* = 37°C; pH = 6.0; *P V* ^−1^ = 11.7 W l^−1^; *F*_gas_ = 5 l h^−1^).

Reconsumption of butyrate and hexanoate by *C. carboxidivorans* was also decreased at low CO partial pressure. Compared with the high CO partial pressure, the concentrations of butanol and hexanol were reduced by a factor of 2.6 and 4.8, respectively (Table 1). Production of the corresponding alcohols is reduced because of restricted water gas shift reaction in *C. carboxidivorans* at low CO concentrations, which results in missing reduction equivalents. If no oxidation of carbon monoxide to carbon dioxide occurs in the methyl branch of the Wood–Ljungdahl pathway, there is no formation of reduced ferredoxin by *C. carboxidivorans*, thereby resulting in a lack of reduction equivalents and decreased capability for reducing fatty acids to the corresponding alcohols (Lee *et al*., 2008). The bifurcating hydrogenase of *C. kluyveri* can as well produce reduced ferredoxin from H_2_ produced by *C. kluyveri* itself as shown in Fig. 2. But it remains an open question whether *C. kluyveri* still forms H_2_ under the given conditions of gassing with CO, since the growth already showed strong restrictions. A net production of hydrogen could not be detected during co‐cultivation (data not shown), so that it cannot be determined whether hydrogen was not produced at all or H_2_ was directly converted. Nevertheless, even if direct conversion of hydrogen took place, the concentration of reduction equivalents present seems to be too low to enable a reduction of the fatty acids to the corresponding alcohols by *C. carboxidivorans*. Additionally, reduced ferredoxin is also essential for creating a proton gradient across the cell membrane of *C. carboxidivorans* by means of the membrane‐bound RNF complex and use of ATPase for generating ATP in acetogenic (Mock *et al*., 2015).

Although the growth of *C. kluyveri* is restricted at an inlet partial pressure of 800 mbar CO at pH 6.0, a remarkable high chain elongation activity was observed in co‐culture, and the organic acids were reduced to the corresponding alcohols by *C. carboxidivorans* resulting in final concentrations of 1.15 ± 0.11 g l^−1^ butyrate/butanol and 0.60 ± 0.04 g l^−1^ hexanoate/hexanol, respectively. This is an increase by a factor of nearly three with respect to the C4‐compounds compared with a mono‐culture of *C. carboxidivorans*, whereby even no hexanoate/hexanol production was observed in the mono‐culture.

A synthetic co‐culture consisting of *C. autoethanogenum* and *C. kluyveri* studied by Diender *et al*. (2016) in anaerobic shaking flasks with a likewise high initially CO partial pressure of 1100 mbar CO in the headspace resulted in final product concentrations of 2.3 g l^−1^ butyrate and 0.7 g l^−1^ hexanoate as well as 0.7 g l^−1^ butanol and 0.4 g l^−1^ hexanol after 12 days. Higher concentrations of the fatty acids were reported after doubling the process time compared with the results of our studies, but 4.9 g l^−1^ acetate was supplemented initially to boost growth of *C. kluyveri* and shaking was omitted for the first 4 days reducing CO diffusion in the liquid phase. With regard to 1‐butanol, we were able to detect higher concentrations after a process time of already 6 days with *C. carboxidivorans* and *C. kluyveri*, which may be attributed to better availability of CO due to the high power input and very much improved gas–liquid mass transfer in the stirred‐tank bioreactor (STR). An extension of the process time would probably also lead to increased product concentrations as shown by Diender *et al*. (2016).

Reducing the inlet partial pressure to 100 mbar CO to allow for improved growth of *C. kluyveri* at pH 6.0 in co‐culture resulted in a severe restriction in the capability of *C. carboxidivorans* to form alcohols. Thus, the organic acids accumulated in co‐culture, and the alcohol‐to‐acid ratios were reduced by a factor of 6.8 and 4.1 for butyrate/butanol and hexanoate/hexanol compared with the batch process at higher inlet CO partial pressure. A constant dying off of *C. carboxidivorans* was also observed.

This shows the importance of convenient process conditions at appropriate CO partial pressures that enable (i) sufficient growth of *C. kluyveri* in co‐culture, (ii) an appropriate C2 product spectrum of *C. carboxidivorans* for chain elongation by *C. kluyveri*, and (iii) a sufficiently high alcohol formation capacity by *C. carboxidivorans*.

Richter *et al*. (2016) studied a synthetic co‐culture of *C. Ijungdahlii* and *C. kluyveri* in a continuously operated stirred‐tank bioreactor with a dilution rate of 0.04 h^−1^ and continuously gassing with a syngas mixture of 60% CO, 35% H_2_ and 5% CO_2_ including *in situ* removal of volatile products by exhaust gas stripping and subsequent condensation. They achieved average steady‐state product concentrations of 0.5 ± 0.1 g l^−1^ butanol and 0.3 ± 0.08 g l^−1^ hexanol in the bioreactor and theoretically 3.3 ± 1.7 g l^−1^ butanol and 2.4 ± 1.3 g l^−1^ hexanol collected in the condensate of the stripped exhaust gas including recycling of the gas with a flow rate of 1.6 l min^−1^ after a process time of 1400 h (~ 58 days) (Richter *et al*., 2016). As we observed comparable concentrations of hexanol and 1.7‐fold increased concentrations of butanol in the liquid phase already after a process time of 144 h in a simple batch process with *C. carboxidivorans* and *C. kluyveri*, continuous operation of a stirred‐tank bioreactor equipped with *in situ* removal of volatile products may not be efficient.

A further option to improve reduction of the organic acids to the corresponding alcohols could be a two‐step process control with an acetogenic phase at pH 6.0 (as already shown in this study) followed by a solventogenic phase (at pH 5.0, for example) for the reduction of the organic acids with *C. carboxidivorans*. This strategy has already been applied with an autotrophic mono‐culture of *C. carboxidivorans* in a continuous process by cascading of two stirred‐tank bioreactors, the first reactor operated at pH 6.0 (acetogenic phase) and the second reactor operated at pH 5.0 (solventogenic phase). Additionally, highly improved steady‐state concentrations of 6.1 g l^−1^ ethanol, 0.7 g l^−1^ butanol and 0.1 g l^−1^ hexanol were achieved in the mono‐culture at a mean hydraulic residence time of 12.5 h (Doll *et al*., 2018). A continuously operated co‐culture of *C. carboxidivorans* and *C. kluyveri* like that may not only be an option to increase the final product concentrations (butanol and hexanol) but the space‐time yield as well.

As the identification of suitable process conditions to enable a more efficient CO‐conversion and formation of C4‐ and C6‐alcohols with *C. carboxidivorans* and *C. kluyveri* in a synthetic co‐culture depends strongly on the individual growth and product formation behaviour of each strain in this co‐culture, this will be successful only by an unbiased individual measurement of the cell concentrations of the two clostridia in co‐culture by FISH‐FC as applied in this study.

## Experimental procedure

### Microorganisms and medium

*Clostridium carboxidivorans* (DSM 15243) and *C. kluyveri* (DSM 555) were purchased from the German Collection of Microorganisms and Cell Culture DSMZ (Braunschweig, Germany). Hurst medium (Hurst and Lewis, 2010) with additional 0.4 g l^−1^ cysteine‐HCl as reducing agent (modified Hurst medium) was used for preculture preparation and the batch studies in stirred‐tank bioreactors (see Supplementary Material). The medium was anaerobically prepared as previously described (Wolfe, 2011; Groher and Weuster‐Botz, 2016).

### Preculture

Each strain received from the DSMZ (Braunschweig, Germany) was first grown in Hurst medium, then supplemented with glycerol and stored as frozen stock culture at −80°C until they were needed for preculture preparation. For preculture preparation, 2.5 ml of a stored frozen stock culture was thawed and then inoculated in anaerobic flasks containing N_2_ in the headspace at a total pressure of 1.0 bar via a septum (butyl rubber stopper, Glasgerätebau Ochs, Bovenden, Germany) using a single‐use syringe (BD Discardit II, Becton Dickinson, Franklin Lakes, USA) and sterile needles (Sterican 0.9 × 70 mm, B. Braun, Melsungen, Germany) as described below:

*Clostridium carboxidivorans* was grown heterotrophically in anaerobic 500 ml flasks with 100 ml modified Hurst medium supplemented with 5 g l^−1^ glucose as carbon source at 37°C in a shaking incubator (Wisecube WIS‐20R, witeg Labortechnik GmbH, Wertheim, Deutschland) at 100 rpm and an eccentricity of 2.5 cm for 21 h.

*Clostridium kluyveri* was grown heterotrophically in anaerobic 500 ml flasks with 100 ml modified Hurst medium with the addition of 10 g l^−1^ potassium acetate and 20 ml l^−1^ ethanol as carbon sources at 37°C and shaking with 100 rpm at an eccentricity of 2.5 cm for 110 h. Concentrations of the carbon sources and the reducing agent were adopted as described in the DSM‐52 medium (DSMZ, Braunschweig, Germany), whereby sodium sulphide was replaced with 0.5 g l^−1^ cysteine‐HCl. 2.5 g l^−1^ Na_2_HCO_3_ was added for providing the HCO_3_/CO_2_ (inorganic carbon) necessary for unimpeded growth of *C. kluyveri* (Tomlinson and Barker, 1954; San‐Valero *et al*., 2019).

Cells were harvested in the exponential growth phase by centrifugation (10 min, 3620 rcf, Hettich Zentrifuga, Rotica 50 RS) and resuspended in anaerobic phosphate‐buffered saline (PBS, pH 7.4) for inoculation of the stirred‐tank bioreactor.

### Stirred‐tank bioreactor and gas fermentation processes

All fermentation processes were carried out in a fully controlled anaerobic 2‐l stirred‐tank reactor (STR) (Labfors, Infors AG, Bottmingen, Switzerland) with a working volume *V*_R_ of 1 l and agitated with two Rushton turbines at 1200 rpm (volumetric power input *P* *V* ^−1^ of 11.7 W l^−1^). Temperature was controlled at 37°C, and pH was kept constant at pH 6.0 by using NaOH (3 M) and H_2_SO_4_ (1 M), respectively. The STR was continuously gassed with gassing rates of 5 Nl h^−1^ (0.083 vvm) and individual gas mixtures of CO:CO_2_:N_2_ controlled by independent mass flow controllers (F‐201CV‐500 RGD‐33‐V, Bronkhorst High‐Tech B.V., Ruurlo, the Netherlands) resulting in defined partial pressures of each gas *p*_i,in_ at the inlet of the bioreactor at a absolute pressure of the system of 1.0 bar. Individual batch processes with pure cultures of *C. kluyveri* and *C. carboxidivorans* were carried out with gas mixtures of 800 mbar N_2_/200 mbar CO_2_ and 800 mbar CO/200 mbar CO_2_, respectively. Studies with varying inlet CO partial pressures p_CO,in_ (0, 100 and 800 mbar, respectively) were achieved by varying the partial pressure of CO and N_2_ as make‐up gas and keeping the partial pressure of CO_2_ constant at *p*_CO2,in_ = 200 mbar.

Autotrophic batch cultivations were carried out by using the modified Hurst medium with 0.5 g l^−1^ cysteine‐HCl as reducing agent. The medium and the bioreactor were autoclaved (121°C, 20 min) prior to inoculation and anaerobized for at least 12 h with the gas mixture under study. Shortly before initiation of the batch experiment, sterile l‐cysteine‐HCl stock solution was added aseptically via a septum (diameter 12 mm, Infors AG, Bottmingen, Switzerland) fixed in the lid of the bioreactor by use of a single‐use syringe (BD Discardit II; Becton Dickinson, Franklin Lakes, NJ, USA) with microfiltration membrane (pore size 0.2 µm, VWR; Radnor, PA, USA) and sterile needles (Sterican 0.9 × 70 mm, B. Braun, Melsungen, Germany) in order to ensure anaerobic conditions and a low redox potential in the stirred‐tank bioreactor. Inoculation was performed by using single‐use syringes (BD Discardit II, Becton Dickinson) and injection through a septum (diameter 12 mm, Infors AG) fixed at the top of the bioreactor to achieve an initial cell dry weight concentration of *c*_x,0_ = 0.05 g l^−1^ in studies with the mono‐cultures. The same initial cell dry weight concentrations were applied in co‐culture studies for each strain resulting in a total initial cell dry weight concentration of *c*_x,0_ = 0.1 g l^−1^.

### Analytical methods

For determination of biomass and product concentrations, 5 ml samples were frequently withdrawn aseptically by sterile single‐use syringes (BD Discardit II, Becton Dickinson) applying sterile needles with a length of 12 cm (Sterican 0.8 × 120 mm, B. Braun) via a septum (diameter 12 mm, Infors AG) fixed at the lid of the stirred‐tank bioreactor.

Product concentrations of organic acids and alcohols were measured by HPLC (Finnigan Surveyor; Thermo Fisher Scientific, Waltham, MA, USA) equipped with a refractive index (RI) detector (Finnigan Surveyor RI Plus Detector; Thermo Fisher Scientific) and an Aminex‐HPX‐87H ion exchange column (Biorad, Munich, Germany). Separation of organic acids and alcohols was carried out at a constant flow rate of 0.6 ml min^−1^ of 5 mM H_2_SO_4_ as eluent at a column temperature of 60°C. Prior to injection, HPLC samples were filtered with a 0.2 μm cellulose filter (Chromafil RC20/15 MS; Macherey‐Nagel GmbH & Co.KG, Düren, Germany). For the measurement of hexanol concentrations, samples were extracted by adding ethyl acetate (sample: ethyl acetate = 4:1), mixing for 15 min at 25 s^−1^ (Retsch MM200; RETSCH GmbH, Haan, Deutschland) and subsequent phase separation by centrifugation at 15 000 rcf for 3 min (Mikro 20; Hettich, Tuttlingen, Germany). The organic phase was then removed and used for HPLC analysis.

A mass flow meter was used for online measurement of the volumetric waste gas flow rate (F‐111B‐1K0‐RGD‐33‐E; Bronkhorst High‐Tech B.V., Ruurlo, the Netherlands). A micro gas chromatograph 490 Micro GC equipped with a 1 m Cox HI column at 80°C and a thermal conductivity detector (Agilent Technologies, Waldbronn, Germany) was applied for the online measurement of CO_2_, CO and H_2_ gas concentrations in the exhaust gas. Nitrogen was used as carrier gas, and a reflux condenser at the top of the stirred‐tank reactor operated at 2°C was used to minimize evaporation of water and alcohols from the bioreactor.

The optical density of the samples at 600 nm (OD_600_) was measured with an UV‐Vis spectrophotometer (Genesys 10S UV‐Vis; Thermo Scientific, Neuss, Germany). For the determination of a correlation factor between OD_600_ and cell dry weight (CDW) concentration, precultures were prepared as described before, concentrated to an optical density OD_600_ of 10 (10 min, 4500 rpm, Hettich Zentrifuga, Rotica 50 RS and resuspended in anaerobic phosphate‐buffered saline (PBS, pH 7.4)). Afterwards, a dilution series was prepared. OD_600_ was measured in technical triplicate for both strains. The cell dry weight (CDW) concentration was measured gravimetrically by drying the centrifuged cell pellet (15 000 rcf, 5 min, Mikro 20; Hettich) in previously dried and weighed 2 mL reaction tubes (Eppendorf AG, Hamburg, Germany) to constant mass at 80°C. The plot of the measured dry biomass concentration versus the OD_600_ enabled the estimation of the linear correlation factors of OD_600_ × 0.48 ± 0.03 g l^−1^ *C. carboxidivorans* and OD_600_ × 0.47 ± 0.04 g l^−1^ *C. kluyveri* (see Fig. S1).

Individual cell dry weight concentrations of *C. carboxidivorans* and *C. kluyveri* in co‐culture were estimated based on the individual cell counts in the sample measured after *in solution* fluorescence *in situ* hybridization (FISH) by flow cytometry (FC). First, each sample was concentrated or diluted with PBS to a final OD_600_ of 0.5, and 0.5 ml was centrifuged at 15 000 rcf (Mikro 20; Hettich). The pellet of each sample was resuspended in 0.5 ml of 50% PBS and 50% ethanol mixture resulting in an OD_600_ of 0.5 and stored at −20°C until further processing.

Steps for *in solution* FISH were adapted and modified from *in situ* FISH to enable cell count measurement via flow cytometry (Lay *et al*., 2007; Haroon *et al*., 2013). Prior to hybridization, the stored samples were dehydrated in a stepwise manner by passaging the cells through 50%, 80% and 98% ethanol–water solutions. The cells were centrifuged for 3 min at 15 000 rcf (Mikro 20; Hettich) after each dehydration step and resuspended in the next solution after discarding the supernatant. The dehydrated bacterial cells were then centrifugated (Mikro 20; Hettich), and the pellet was resuspended and permeabilized by adding 0.5 ml of a 20% lysozyme solution (10 g l^−1^) for 10 min on ice followed by centrifugation for 5 min at 15 000 rcf (Mikro 20; Hettich). After washing of the cells once in deionized water, the cells were resuspended in 0.5 ml hybridization buffer containing 30% formamide and 0.5 µg ml^−1^ of each strain‐specific 23S rRNA‐targeted oligonucleotide probe (see Supplementary Material) and incubated for 3.5 h at 46°C. The 23S rRNA‐targeted oligonucleotide probes ClosKluy and ClosCarb were designed using the Arb software package (Ludwig *et al*., 2004) together with the Silva database (Quast *et al*., 2013). Optimal formamide concentration and specificity of the designed probes were verified by cross‐hybridization experiments and with different reference strains of the genus *Clostridium* (Schneider *et al*., 2021). Non‐bound oligonucleotide probes were removed by washing the cells in 0.5 ml of preheated washing buffer (see Supplementary Material) twice including incubation at 48°C for 20 min. Finally, the bacterial cells were washed with ice‐cold PBS twice and resuspended in a final volume of 0.5 ml PBS.

A flow cytometer (BD FACS Melody Cell Sorter; BD Biosciences, San Jose, CA, USA) equipped with a blue and red laser exciting at 488 nm and 640 nm was used for the individual determination of the cell counts. The rate of events was set to 10 000 events s^−1^ and analysed using the BD FACSChorus software (BD Biosciences). The side angle scatter (SSC) and the forward angle scatter (FSC) were measured with the blue laser emitting at 488 nm by applying the 488‐nm band‐pass filter. The fluorescence signal of the fluorescein isothiocyanate (FITC) labelled probes of *C. kluyveri* was excited at 488 nm, and the emission of FITC was measured applying the 527/32‐nm band‐pass filter (FITC filter set). The cyanine 5 (Cy5)‐labelled probes of *C. carboxidivorans* were excited by the red laser at 640 nm, and emission of Cy5 was measured applying the 660/10‐nm band‐pass filter (allophycocyanin‐cyanine 7 (APC‐C7) filter set). The acquisition threshold was set in the side scatter channel in order to reduce background noise and exclude the detection of particular PBS components. Fluorescence signals of the cells were collected and plotted as scattergrams (SSC vs. FSC) of the fluorescence intensity with logarithmic display and as histograms with cell counts vs. the fluorescence intensity in the corresponding filter set in bi‐exponential display. Cells of each strain were counted by the software using the prior defined gates. The OD_600_ of each strain was then obtained by using the specific linear correlation factors between cell counts and OD_600_, which were determined before by monitoring the cell counts in manually mixed samples with different combinations of both strains characterized by their OD_600_ and summed up to a total OD_600_ of 0.5. The so determined linear correlation factors were cell counts (per 10 000 events) × 5.1 ± 0.03 × 10^−5^ for OD_600_ of *C. kluyveri* and cell counts (per 10 000 events) × 5.3 ± 0.1 × 10^−5^ for OD_600_ of *C. carboxidivorans* (see Fig. S2). Unlabelled samples were measured as negative control. The CDW concentrations of both strains were then calculated by applying the linear correlation factors between CDW and OD_600_ (see above).

## Conflict of interest

None declared.

## Supporting information


**Fig. S1.** Determination of the strain specific linear correlation factor of *C. kluyveri* (A) and *C. carboxidivorans* (B). For this purpose, precultures were concentrated to an OD_600_ of 7 and a dilution series was prepared. OD_600_ and the gravimetrically determined CDW concentration were measured in triplicate. The linear correlation factors were OD_600_ × 0.47 ± 0.04 g l^−1^ for *C. kluyveri* and OD_600_ × 0.48 ± 0.03 g l^−1^ for *C. carboxidivorans*.
**Fig. S2.** Specific linear correlation factor between cell counts and OD_600_, which were estimated to cell counts (per 10 000 events) × 5.1 ± 0.03 × 10^−5^ for OD_600_ of *C. kluyveri* (A) and cell counts (per 10 000 events) × 5.3 ± 0.1 × 10^−5^ for OD_600_ of *C. carboxidivorans* (B) by measuring the cell counts with the flow cytometer at varying OD_600_ of each individual strain summing up to a final OD_600_ of 0.5 of the mixture. Unlabeled samples were measured as negative controls.
**Fig. S3.** Scattergrams with the intensity of the fluorescence signals of *C. carboxidivorans* in red (A) and *C. kluyveri* in green (B). Individual samples of pure cultures of *C. carboxidivorans* and pure cultures of *C. kluyveri* were mixed with the strain specific oligonucleotide probes and the resulting cell numbers were measured with flow cytometry. Blue dots indicated other, non‐labeled particles. The axes show the intensity of the fluorescence signal (arbitrary units) (A = Area).
**Table S1.** Strain specific 23S rRNA targeted oligonucleotide probe.
**Table S2.** Modified Hurst medium (Hurst & Lewis, 2010) used for preculture and batch studies.
**Table S3.** Hybridization and washing buffer used for *in solution* FISH.
