# Supplementary material for: Synthetic co‐culture of autotrophic Clostridium carboxidivorans and chain elongating Clostridium kluyveri monitored by flow cytometry
Source: Microb Biotechnol. 2021 Oct 20;15(5):1471–85. doi: 10.1111/1751-7915.13941 (PMC9049614; doi:10.1111/1751-7915.13941)
Supplement: Supplementary file 1 — Fig. S1. Determination of the strain specific linear correlation factor of C. kluyveri (A) and C. carboxidivorans (B). For this purpose, precultures were concentrated to an OD600 of 7 and a dilution series was prepared. OD600 and the gravimetrically determined CDW concentration were measured in triplicate. The linear correlation factors were OD600 × 0.47 ± 0.04 g l−1 for C. kluyveri and OD600 × 0.48 ± 0.03 g l−1 for C. carboxidivorans. Fig. S2. Specific linear correlation factor between cell counts and OD600, which were estimated to cell counts (per 10 000 events) × 5.1 ± 0.03 × 10^−5^ for OD600 of C. kluyveri (A) and cell counts (per 10 000 events) × 5.3 ± 0.1 × 10^−5^ for OD600 of C. carboxidivorans (B) by measuring the cell counts with the flow cytometer at varying OD600 of each individual strain summing up to a final OD600 of 0.5 of the mixture. Unlabeled samples were measured as negative controls. Fig. S3. Scattergrams with the intensity of the fluorescence signals of C. carboxidivorans in red (A) and C. kluyveri in green (B). Individual samples of pure cultures of C. carboxidivorans and pure cultures of C. kluyveri were mixed with the strain specific oligonucleotide probes and the resulting cell numbers were measured with flow cytometry. Blue dots indicated other, non‐labeled particles. The axes show the intensity of the fluorescence signal (arbitrary units) (A = Area). Table S1. Strain specific 23S rRNA targeted oligonucleotide probe. Table S2. Modified Hurst medium (Hurst & Lewis, 2010) used for preculture and batch studies. Table S3. Hybridization and washing buffer used for in solution FISH. [file MBT2-15-1471-s001.docx]

**Supplementary material.**

**Figure 1**

**
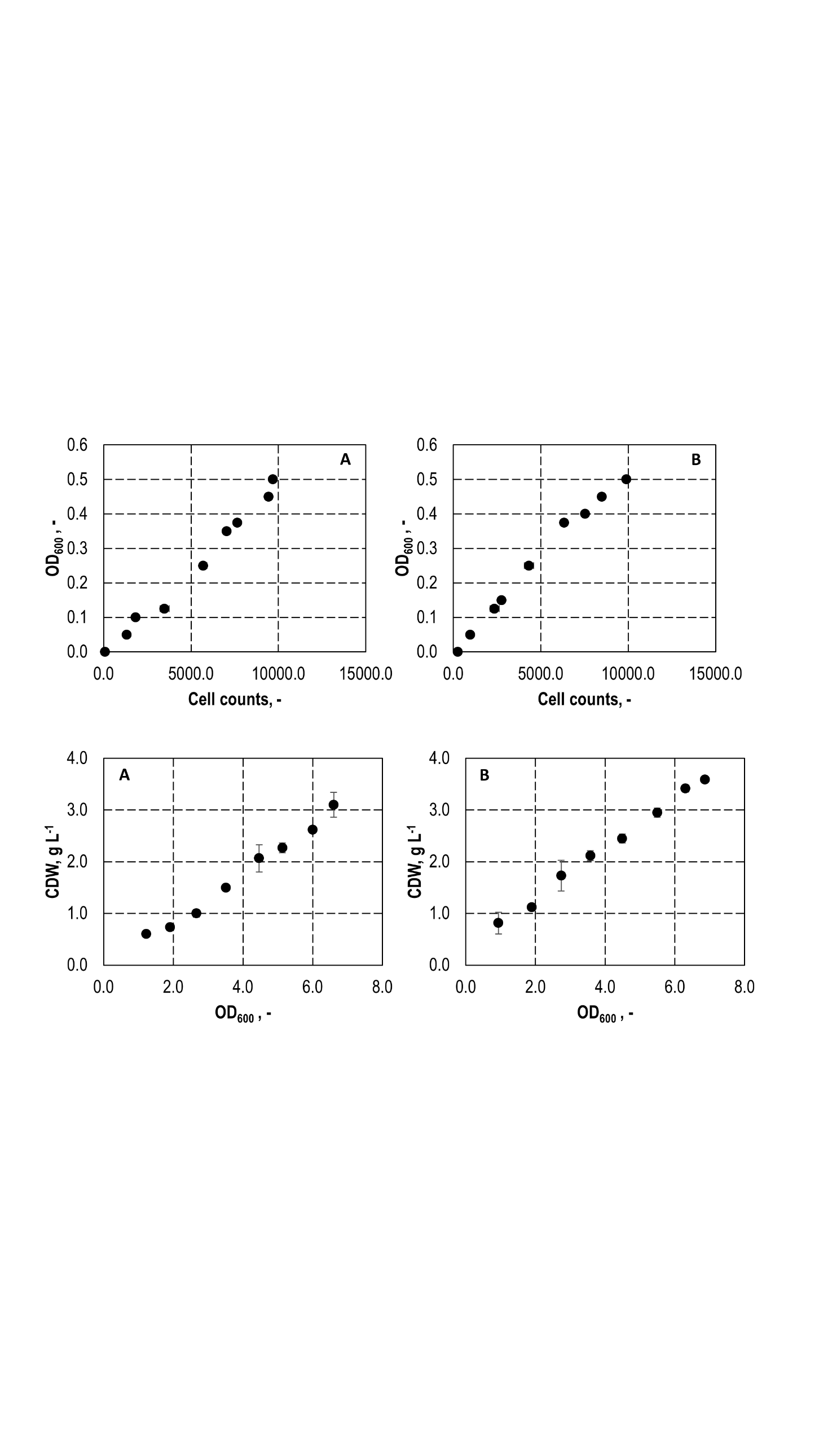
**

**Figure S 1** Determination of the strain specific linear correlation factor of *C. kluyveri* (A) and *C. carboxidivorans* (B). For this purpose, precultures were concentrated to an OD_600_ of 7 and a dilution series was prepared. OD_600_ and the gravimetrically determined CDW concentration were measured in triplicate. The linear correlation factors were OD_600_ × 0.47 ± 0.04 g L^−1^ for *C. kluyveri* and OD_600_ x 0.48 ± 0.03 g L^−1^ for *C. carboxidivorans*.

**
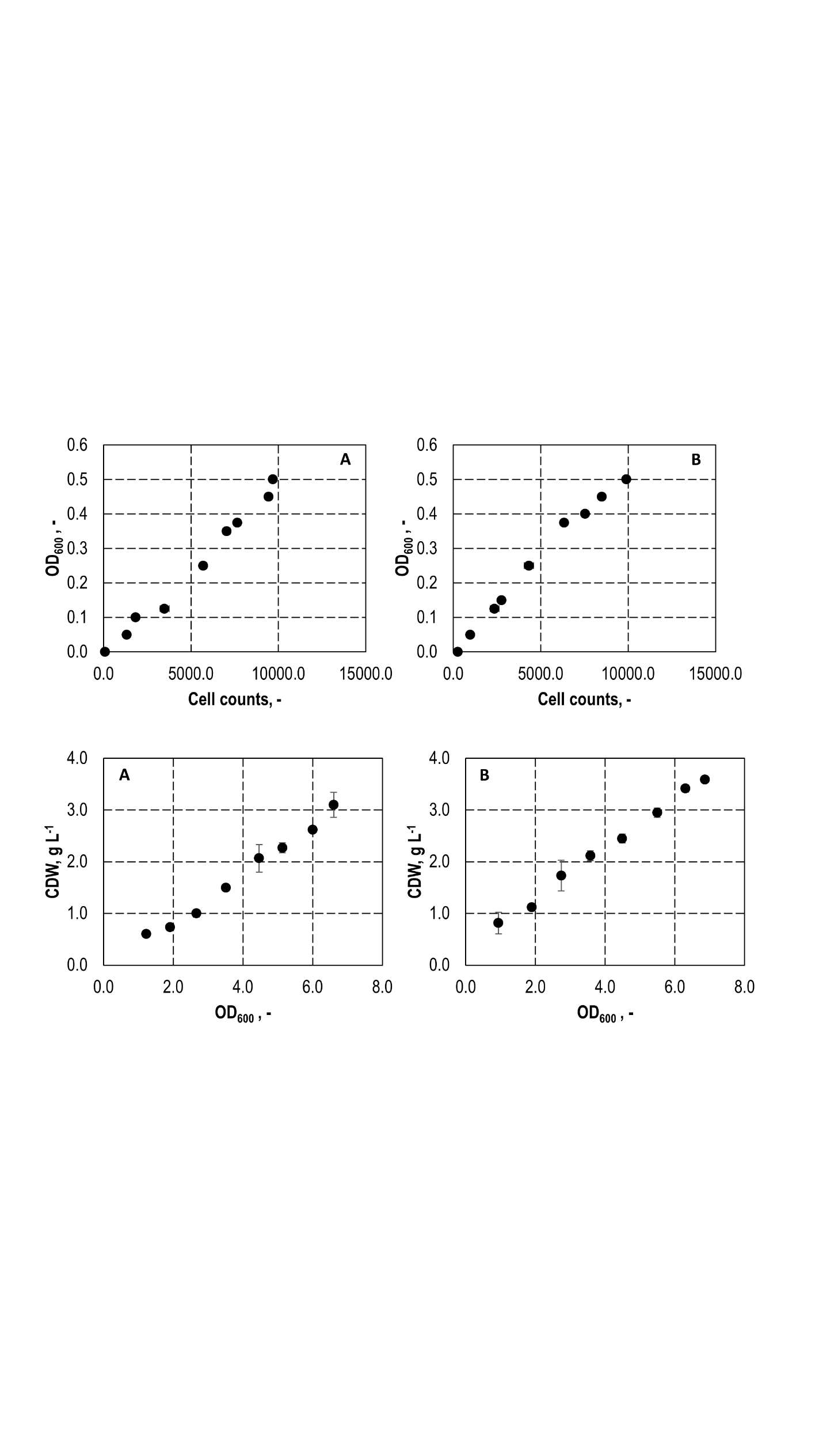
**

**Figure S 2** Specific linear correlation factor between cell counts and OD_600_, which were estimated to cell counts (per 10,000 events) × 5.1 ± 0.03 × 10^−5^ for OD_600_ of *C. kluyveri* (A) and cell counts (per 10,000 events) × 5.3 ± 0.1 × 10^−5^  for OD_600_ of *C. carboxidivorans* (B) by measuring the cell counts with the flow cytometer at varying OD_600_ of each individual strain summing up to a final OD_600_ of 0.5 of the mixture. Unlabeled samples were measured as negative controls.


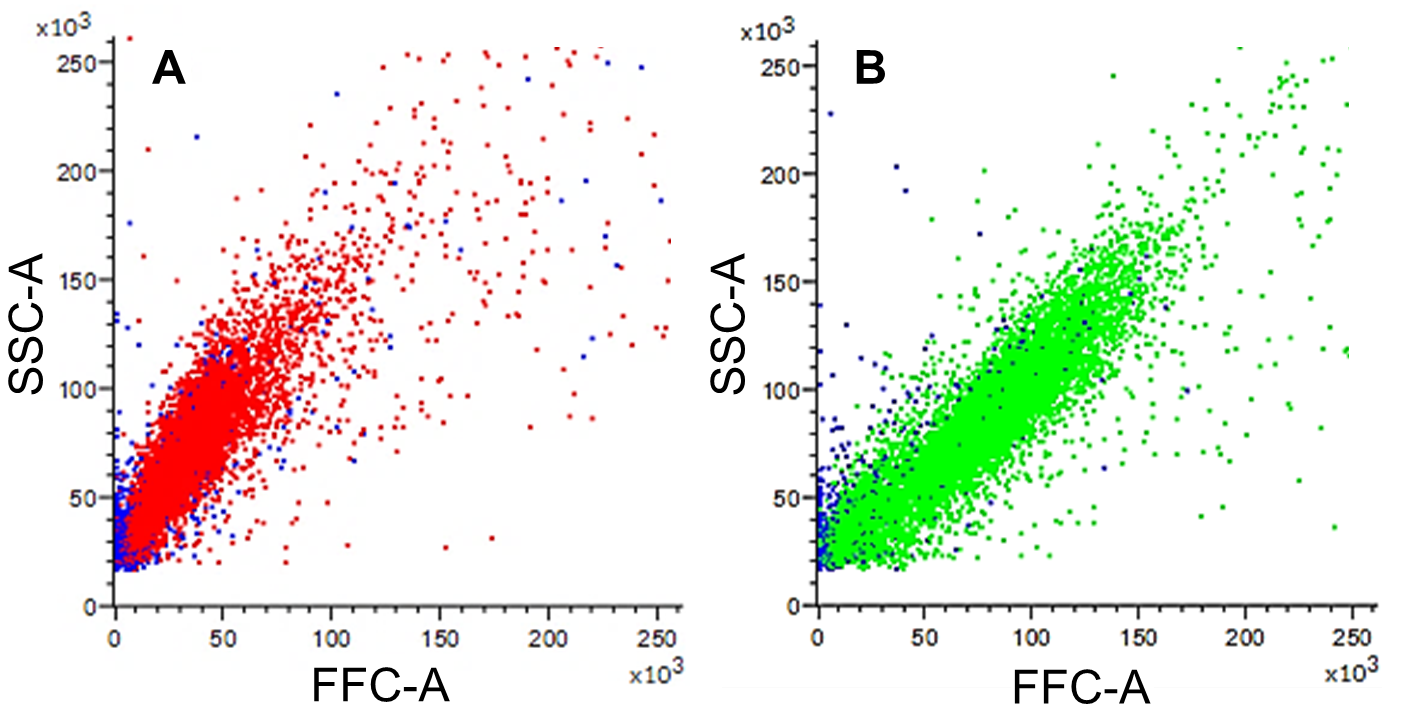


**Figure S 3** Scattergrams with the intensity of the fluorescence signals of *C. carboxidivorans* in red (A) and *C. kluyveri* in green (B). Individual samples of pure cultures of *C. carboxidivorans* and pure cultures of *C. kluyveri* were mixed with the strain specific oligonucleotide probes and the resulting cell numbers were measured with flow cytometry. Blue dots indicated other, non-labeled particles. The axes show the intensity of the fluorescence signal (arbitrary units) (A = Area).

**Tables**

**Table S 1** Strain specific 23S rRNA targeted oligonucleotide probe

**Name Sequence 5′ 🡪 3′ Fluorophore T_m_ [C°] G+C [%]**

ClosKluy_1516 GCGGACTCCCCTTCAAAG FITC 58 61

ClosCarb_1516 AGCCACTCCCCATCACAC Cy5 58 61

**Table S 2** Modified Hurst medium (Hurst & Lewis, 2010) used for preculture and batch studies

**Component Formula Concentration in Stock solution, g L^−1^**

**Mineral solution 33.3x**

Ammonium chloride NH_4_Cl 100

Sodium chloride NaCl 80

potassium chloride KCl 10

potassium dihydrogen phosphate KH_2_PO_4_ 10

Magnesium sulfate MgSO_4_ 20

Calcium chloride CaCl_2_ 4

**Vitamin solution 100x**

Pyridoxine C_8_H_11_NO_3_ 0.01

Thiamine C_12_H_17_ClN_4_OS 0.005

Riboflavin C_17_H_20_N_4_O_6_  0.005

Calcium pantothenate Ca(C_9_H_16_NO_5_)_2_ 0.005

Liponic acid C_8_H_14_O_2_S_2_  0.005

Para amino benzoic acid C_7_H_7_NO_2_ 0.005

Nicotinic acid C_6_H_5_NO_2_ 0.005

Vitamin B12 C_72_H_100_CoN_18_O_17_P 0.005

D-biotin C_10_H_16_N_2_O_3_S 0.002

Folic acid C_19_H_19_N_7_O_6_ 0.002

2-mercapto ethane sulfonic acid C_2_H_6_O_3_S_2_ 0.02

**Trace element solution 100x**

Nitrilotriacetic acid C_6_H_9_NO_6_ 2.00

Manganese sulfate MnSO_4_ 1.00

Ammonium iron sulfate NH_4_Fe(SO_4_)_2_ 0.80

Cobalt chloride CoCl_2_ 0.20

Zinc sulfate ZnSO_4_  0.20

Copper chloride CuCl_2_ 0.02

Nickel chloride NiCl_2_ 0.02

Sodium molybdate Na_2_MoO_4_ 0.02

Sodium selenate Na_2_SeO_4_ 0.02

Sodium wolframate Na_2_WO_4_ 0.02

**Medium concentration**

**Yeast extract** 1.0 g L^−1^

**Cysteine hydrochloride** C_3_H_7_NO_2_S HCl 0.4–0.5 g L^−1^

**Morpholino ethanesulfonic acid^a)^** C_6_H_13_NO_4_S 15.0 g L^−1^

**Polypropylene glycol (1:10)^b)^** 0.1 mL L^−1^

a) used only for heterotrophic preculture in anaerobic flasks

b) only for reactor studies

**Table S 3** Hybridization and washing buffer used for *in solution* FISH

**Component Hybridization buffer Washing buffer Unit**

NaCl (5 M) 180 20.4 mL L^−1^

Tris-HCl (1 M) 20 20 mL L^−1^

DI-H_2_0 500 948.6 mL L^−1^

Formamide 300 - mL L^−1^

EDTA (0.5 M) - 10 mL L^−1^

SDS (10% w/v) 1 1 mL L^−1^
